# Prevalence and associated factors of adult overweight and obesity in Southwestern China

**DOI:** 10.3389/fpubh.2025.1507467

**Published:** 2025-02-12

**Authors:** Xiao-Qiang Zhang, Hua-An Du, Chuan Huang, Jian-Xiong Liu, Yong-Mei Hu, Ya Liu, Xiao-Bo Huang

**Affiliations:** ^1^Division of Cardiology, Chengdu Second People’s Hospital, Chengdu, Sichuan, China; ^2^Division of Cardiology, University-Town Hospital of Chongqing Medical University, Chongqing, China; ^3^Division of Endocrinology and Metabolism, Chengdu Second People’s Hospital, Chengdu, Sichuan, China

**Keywords:** overweight, obesity, prevalence, risk factors, China

## Abstract

**Background:**

Data on the prevalence of overweight and obesity in Southwestern China were limited. The aims of this study were to estimate the prevalence of overweight/obesity and their associated factors in this area.

**Methods:**

A cross-sectional study was conducted from 2013 to 2014 in Chengdu and Chongqing, two megacities in Southwestern China. Data were obtained from questionnaires, physical examinations and lab tests. A total of 11,096 residents aged 35–79 years were included in the final analysis of this study.

**Results:**

The prevalence of overweight and obesity among adults aged 35–79 years in Southwestern China were 29.7 and 4.4%, respectively. Multivariable logistic regression analysis suggested that women, non-smokers, ex-smokers, being hypertensive and diabetic were related to higher obesity prevalence, and that physically active adults and those aged 65–79 years were less likely to have obesity.

**Conclusion:**

Obesity and overweight were prevalent in Southwestern China, especially among women, those with diabetes and/or hypertension, and those who have quitted smoking for more than 3 years.

## 1 Introduction

Overweight and obesity have increased in pandemic dimensions (1–5). To make the situation more complexing, regional differences exist in both obesity prevalence and trends between countries and within countries (1–4). Data (2) show that higher-than-optimal BMI caused an estimated 5 million deaths from noncommunicable diseases such as cardiovascular diseases, diabetes, cancers, neurological disorders, chronic respiratory diseases, and digestive disorders in 2019. To address the rising pandemic of obesity, there are widespread calls for regular monitoring of the trends in adult overweight and obesity prevalence in all populations (3–5). The prevalence of overweight and obesity in China have been on the rise in recent decades (6–8) and are projected to increase further (9), primarily driven by an increasing adoption of a western lifestyle and decreased physical activity (10, 11). Literature consistently shows that the prevalence of overweight and obesity varied considerably among provinces and regions in China (6–8). More than 14% of China’s population live in Southwestern China (12), where about 53 million living in two megacities - Chengdu and Chongqing (13, 14). Southwestern China has economically developed fast over the past few decades, resulting in dramatic changes of lifestyle and probably growing prevalence of overweight and obesity. However, data on the prevalence and related factors of adult overweight and obesity in this area were lacking. In the current article, we estimate the prevalence of adult overweight and obesity and explore their potential influencing factors in Southwestern China.

## 2 Methods

### 2.1 Study oversight

Data used in our study were obtained from the survey of cardiovascular risk factors in Chengdu and Chongqing performed from 2013 to 2014. The survey was conducted in accordance with the basic principles of the Declaration of Helsinki. The protocol of this survey was approved by Ethics Committee of the Chengdu Second People’s Hospital (No 2013015). All participants provided written informed consent before the enrollment.

### 2.2 Study population

This was a population-based cross-sectional survey conducted in urban areas of Chengdu and Chongqing, Southwestern China from September 2013 to March 2014. A multi-stage sampling method was used to select the study sample. In the first stage, we randomly selected three districts of Jinjiang, Longquanyi and Chenghua from Chengdu City, and two districts of Yubei and Jiangbei from Chongqing City. In the second stage, a subdistrict was selected randomly from each of these five districts. The third stage involved a random selection of one community from each subdistrict. Residents aged 35–79 years old were eligible if they had lived in these communities for 5 years or longer. According to the protocol, residents were ruled out if they had mental illness, malignancy, secondary hypertension, or if they were on dialysis or refused to participate.

### 2.3 Data collection

The current survey included face-to-face interviews, physiological examinations, both administered by trained medical personnel, and as well as laboratory tests. The health interview involved questions on demographic characteristics, lifestyle habits and other health-related questions. Examination components included body measurements (height and weight) and blood pressure. Height was measured without shoes and hat. Weight was measured after removal of shoes, hat and heavier trousers. The blood pressure was measured twice and the mean value was recorded. Laboratory components included fasting plasma glucose (FPG) and 2-h plasma glucose (2-h PG) after a 75 g oral glucose.

BMI was calculated as weight in kilograms divided by the square of the height in meters. Overweight and obesity were, respectively, defined as 25 ≤ BMI < 30 kg/m^2^ and BMI ≥ 30 kg/m^2^ according to the WHO classification (15); the BMI cut-off points of the Working Group on Obesity in China (BMI ≥ 24.0 kg/m^2^, BMI ≥ 28.0 kg/m^2^) for overweight and obesity were also used (16). Smokers were defined as having smoked at least one cigarette per day and for more than a year, currently or having no smoking for less than 3 years prior to the survey. Ex-smokers were defined as those quitted smoking 3 years ago or earlier and once having at least one cigarette per day and for more than a year. Non-smokers were defined as having never smoked or having an average of less than one cigarette daily for less than a year. Alcohol drinkers were defined as having consumed at least once a week and for more than a year, currently or having no drinking for less than 3 years. Ex-drinkers were defined as those quitted drinking alcohol 3 years ago or earlier and once consumed alcohol at least once a week and for more than a year. Non-drinkers were defined as having never consumed alcohol or having consumed an average of less than once a week for less than a year. Participants were considered as being physically active if they reported having physical activity at least once a week. Patients were considered to have diabetes if they had a FPG level of at least 7.0 mmol/L and/or a 2-h PG level of at least 11.1 mmol/L, or if they had a medical history of diabetes. Patients were considered to have hypertension if they had a systolic blood pressure value of at least 140 mmHg and/or a diastolic blood pressure of at least 90 mmHg, or if they had a medical history of hypertension.

### 2.4 Statistical analysis

All statistical analyses were conducted with SPSS 23.0 software. A *p* value of <0.05 was considered to be statistically significant. Continuous variables were presented as the mean ± standard derivation (SD), and the differences between different groups were compared by t-test or analysis of variance. Categorical variables were presented as percentage (%), and chi-square test was used to compare the differences between different groups. Trend analysis was done by Chi-Square trend test. The univariable and multivariable analyses of obesity were conducted by using the non-conditional Logistic regression model, and variable selecting was conducted by using the forward stepwise selection method. The likelihood ratio (LR) and odds ratio (OR) value and its 95% confidence interval were calculated.

## 3 Results

We excluded 638 participants from the 14,016 eligible residents according to the protocol and then 2,282 participants due to incomplete information, resulting in a total of 11,096 participants included in the final analysis. The mean age of the participants was 55.09 ± 10.96 years, 91.0% were married, 64.5% were women, 23.7% were self-reported having high school education or higher, 58.9% were physically active; 22.3% were smokers, 75.1% were non-smokers and 2.6% were ex-smokers; 17.2% were alcohol drinkers, 81.3% were non-drinkers and 1.5% were ex-drinkers. The mean values of systolic and diastolic blood pressure were 130.92 ± 21.53 mmHg and 78.50 ± 11.38 mmHg, respectively. The mean FPG and 2-h PG levels were 5.67 ± 2.05 mmol/L and 7.92 ± 3.83 mmol/L, respectively.

Compared with men (Table 1), women appeared to have higher rates of non-smokers (95.3% vs. 38.4%, *p* < 0.001), alcoholic non-drinkers (95.5% vs. 55.4%, *p* < 0.001) and being physically active (60.0% vs. 57.0%, *p* = 0.002), and have a lower rate of being married (88.8% vs. 95.0%, *p* < 0.001), having lower education (19.0% vs. 32.2%, *p* < 0.001). Women had significantly lower mean systolic and mean diastolic blood pressure values (129.90 ± 21.96 mmHg vs. 132.77 ± 20.08 mmHg, *p* < 0.001 and 77.45 ± 11.37 mmHg vs. 80.40 ± 11.14 mmHg, *p* < 0.001, respectively) and a significantly higher mean 2-h PG level (8.01 ± 3.83 mmol/L vs. 7.75 ± 3.82 mmol/L, *p* = 0.001). No significant difference for the FPG level was observed between male and female (5.66 ± 2.14 mmol/L vs. 5.69 ± 1.89 mmol/L, *p* = 0.513).

**Table 1:** Characteristics of the participants stratified by sex^*^.

Variable	Overall (*n* = 11,096)	Men (*n* = 3,935)	Women (*n* = 7,161)	*p* values
Age (y)	55.09 ± 10.96	56.36 ± 11.21	54.39 ± 10.75	< 0.001
Married (%)	10,097 (91.0)	3,738 (95.0)	6,359 (88.8)	< 0.001
Higher education^†^ (%)	2,629 (23.7)	1,269 (32.2)	1,360 (19.0)	< 0.001
Smoking status (%)				< 0.001
Smoker	2,472 (22.3)	2,175 (55.3)	297 (4.1)	
Non-smoker	8,338 (75.1)	1,510 (38.4)	6,828 (95.3)	
Ex-smoker	286 (2.6)	250 (6.4)	36 (0.5)	
Alcohol drinking status (%)				< 0.001
Drinker	1911 (17.2)	1,631 (41.4)	280 (3.9)	
Non-drinker	9,021 (81.3)	2,181 (55.4)	6,840 (95.5)	
Ex-drinker	164 (1.5)	123 (3.1)	41 (0.6)	
Physically active (%)	6,505 (58.9)	2,231 (57.0)	4,274 (60.0)	0.002
SBP (mmHg)	130.92 ± 21.35	132.77 ± 20.08	129.90 ± 21.96	< 0.001
DBP (mmHg)	78.50 ± 11.38	80.40 ± 11.14	77.45 ± 11.37	< 0.001
FPG (mmol/L)	5.67 ± 2.05	5.69 ± 1.89	5.66 ± 2.14	0.513
2-h PG (mmol/L)	7.92 ± 3.83	7.75 ± 3.82	8.01 ± 3.83	0.001

^*^SBP denotes systolic blood pressure, DBP denotes diastolic blood pressure, FPG denotes fasting plasma glucose, 2-h PG denotes 2-h plasma glucose.

^†Participants reported having high school education or higher.^

In the overall participants, the mean BMI was 23.90 ± 3.53 kg/m^2^ (Table 2). One-way ANOVA analysis showed a significant difference across the age groups (*p* < 0.05). Additionally, women (24.04 ± 3.60 kg/m^2^) had a higher BMI than men (23.64 ± 3.39 kg/m^2^) (*p* < 0.05). As for overweight and obesity (Table 3), the prevalence were 29.7 and 4.4%, respectively. The prevalence of overweight increased significantly with advancing age (*P* for trend <0.05). Comparing with men, women have a significantly higher prevalence of overweight (30.5% for women and 28.1% for men, *p* < 0.05) and obesity (5.1% for women and 3.2% for men, *p* < 0.05). Similar results were observed based on the BMI cutoffs recommended by the Working Group on Obesity in China (Table 4).

**Table 2:** Age-sex-specific distributions of BMI^*^.

Age	Overall (*n* = 11,096)	Men (*n* = 3,935)	Women (*n* = 7,161)
35–44	23.70 ± 3.56	23.67 ± 3.34	23.71 ± 3.66
45–54	23.78 ± 3.57	23.66 ± 3.30	23.83 ± 3.68
55–64	24.16 ± 3.49	23.74 ± 3.44	24.41 ± 3.50
65–79	23.85 ± 3.47^†^	23.49 ± 3.42	24.13 ± 3.48
Total	23.90 ± 3.53	23.64 ± 3.39^‡^	24.04 ± 3.60

^*^BMI denotes body-mass index.

^†^One-way analysis of variance (ANOVA) *p* < 0.05.

^‡Compared with women, p < 0.05.^

**Table 3:** Age-sex-specific prevalence of overweight and obesity defined by WHO.

Age	Overall (*n* = 11,096)		Men (*n* = 3,935)		Women (*n* = 7,161)	
	Overweight	Obesity	Overweight	Obesity	Overweight	Obesity
35–44	668 (27.0)	106 (4.3)	231 (28.5)	27 (3.3)	437 (26.3)	79 (4.8)
45–54	803 (29.1)	117 (4.2)	233 (29.2)	16 (2.0)	570 (29.0)	101 (5.1)
55–64	1,071 (30.1)	177 (5.0)	359 (27.2)	46 (3.5)	712 (31.7)	131 (5.8)
65–79	748 (32.6)^†^	88 (3.8)	282 (28.0)	35 (3.5)	466 (36.1)	53 (4.1)
Total	3,290 (29.7)	488 (4.4)	1,105 (28.1)^*^	124 (3.2)^‡^	2,185 (30.5)	364 (5.1)

^†p-trend for age < 0.05.^

^*^Compared with women, *p* < 0.05.

^‡Compared with women, p < 0.05.^

**Table 4:** Age-sex-specific prevalence of overweight and obesity defined by the Working Group on Obesity in China.

Age	Overall (*n* = 11,096)		Men (*n* = 3,935)		Women (*n* = 7,161)	
	Overweight	Obesity	Overweight	Obesity	Overweight	Obesity
35–44	752 (30.4)	274 (11.1)	288 (35.5)	69 (8.5)	464 (28.0)	205 (12.4)
45–54	925 (33.5)	295 (10.7)	279 (35.0)	68 (8.5)	646 (32.9)	227 (11.5)
55–64	1,328 (37.3)	413 (11.6)	462 (35.0)	115 (8.7)	866 (38.6)	298 (13.3)
65-	850 (37.0)^†^	239 (10.4)	343 (34.1)	79 (7.8)	507 (39.3)	160 (12.4)
Total	3,855 (34.7)	1,221 (11.0)	1,372 (34.9)	331 (8.4)^‡^	2,483 (34.7)	890 (12.4)

^†p-trend for age < 0.05.^

^‡Compared with women, p < 0.05.^

Univariable logistic regression analysis suggested that women, non-smokers, ex-smokers, non-drinkers, being hypertensive and diabetic were associated with a higher prevalence of obesity and that being physically active was associated with a lower obesity prevalence (Table 5). Multivariable logistic regression analysis further supported that women, non-smokers, ex-smokers, being hypertensive and diabetic were related to higher obesity prevalence, and that physically active adults and those aged 65–79 years were less likely to have obesity.

**Table 5:** Univariable and multivariable logistic regression analysis of obesity and the associated factors.

	Univariable model	Multivariable model
Sex		
Men	1.000 (reference)	1.000 (reference)
Women	1.646 (1.337–2.026)^†^	1.352 (1.035–1.765)^†^
Age (years)		
35–44	1.000 (reference)	1.000 (reference)
45–54	0.986 (0.754–1.290)	0.941 (0.718–1.233)
55–64	1.165 (0.911–1.491)	0.989 (0.768–1.274)
65–79	0.888 (0.665–1.185)	0.658 (0.487–0.889)^†^
Married	0.948 (0.695–1.294)	
Higher education^*^	1.179 (0.773–1.796)	
Smoking history		
Smoker	1.000 (reference)	1.000 (reference)
Non-smoker	1.871 (1.437–2.436)^†^	1.485 (1.074–2.055)^†^
Ex-smoker	2.018 (1.136–3.584)^†^	1.958 (1.096–3.497)^†^
Alcohol drinking history		
Drinker	1.000 (reference)	
Non-drinker	1.572 (1.189–2.078)^†^	
Ex-drinker	1.424 (0.639–3.173)	
Physically active	0.698 (0.552–0.884)^†^	0.752 (0.589–0.960)^†^
Hypertension	1.981 (1.651–2.377)^†^	1.987 (1.641–2.406)^†^
Diabetes mellitus	1.593 (1.299–1.952)^†^	1.401 (1.135–1.731)^†^

^†p < 0.05.^

^*^Participants reported having high school education or higher.

## 4 Discussion

To our knowledge, this is the first large population-based face to face survey to investigate the prevalence of overweight and obesity in Chengdu and Chongqing in Southwestern China. The current study shows that 34.1% of the adults in this area had overweight or obesity. However, the obesity prevalence among the Southwestern China adults in our study (4.4%) is lower than the national prevalence of obesity in China (5.2%) (7), much lower than the adult obesity prevalence in the United States (41.9%) (17), that in Asian Americans (16.1%) (17), and that in the European Region (23%) (18), there are estimated to be several million adults with a BMI of ≥30 kg/m^2^ due to the large population in this area. Furthermore, a recent research (9) suggests that the prevalence of overweight and obesity in Chinese adults are projected to increase further. Unsurprisingly, obesity and overweight represent major health challenges for local health-care system.

The prevalence of overweight and obesity in Southwestern China in our study were comparable to those in regions of South China (19) but much lower than those in North China (20–23). The striking North–South difference of the obesity prevalence in China has been documente with a BMI of ≥30 kg/m^2^ d before (6–8). The regional disparities of obesity prevalence within countries have also been reported in Germany (24) and in the United States (25). The regional differences between North and South China are at least partly related to or resulted from differences in climate, diet and living habits between the two areas (26, 27).

Obesity is a complex condition with many causal contributors including genetic ones and many environmental factors (28). Unsurprisingly not only the prevalence but also the associated risk factors of obesity vary across different regions of China. Our study shows that BMI, overweight prevalence and obesity prevalence in women were significantly higher than in men, in line with the findings of some researches in China (21). Multivariate logistic regression analysis suggested that women were related to a higher obesity prevalence. However, large heterogeneity in obesity prevalence in women and men existed in the published literature. A higher obesity prevalence in women than in men was also found at the global level (1). However, a lower obesity prevalence among women than among men was reported in some provinces of China (22, 23). In a research conducted in the overall China (8), men had a lower prevalence of obesity than did women in 2004, but this pattern had reversed by 2018. In the United States, there was no significant differences of adult obesity prevalence between men and women although the prevalence of severe obesity in adults was higher in women than men (17). The disparities suggest women may not be an independent risk factor of obesity, and understanding the drivers of these disparities might help to provide guidance for the most promising intervention strategies.

The current study shows the prevalence of obesity increased progressively with increasing age until 65 years, then dropped after 65 years, similar to previous studies (6), while the prevalence of overweight grew with increasing age across all age groups. The definite cause of the obesity prevalence drop in the older adult seems elusive. An assumption in a previous study (6) was that it was due to a decrease in total energy intake, muscle loss with age, chronic disease consumption in the older adult (29, 30). However, this assumption failed to explain the increased prevalence of overweight in the adults aged of 65–79 years. Another theory is that it could be due to survival disadvantage associated with obesity in the older population (28), which could explain the paradoxical changes of prevalence of overweight and obesity in the age group 65–79 since in older adults, as a meta-analysis showed (31), being overweight was not found to be associated with an increased mortality risk while having a BMI >33 kg/m^2^ was.

Our study shows ex-smokers as well as non-smokers were more likely to have obesity, consistent with prior study (19). Smoking cessation at every age was associated with longer survival (32) and undoubtedly should be encouraged for all smokers even in the presence of a potential risk of weight gain. However, professional advice for weight control is reasonable for a greater health benefit even after 3 years of smoking cessation.

There are several limitations in this study. Firstly, the participants in our study were enrolled from Chengdu and Chongqing which made it inappropriate to extrapolate its conclusion to other regions of China. Secondly, the survey was conducted in urban areas of Southwestern China. As a result, it should be cautious to extrapolate the results to rural areas although recent evidence showed narrowing urban–rural difference among men and even higher mean BMI and obesity prevalence in women in rural settings compared with women in urban settings (8). Thirdly, the causal relationships between overweight/obesity and their associated factors cannot be established due to the cross-sectional design of the current study.

## 5 Conclusion

In summary, our study provides important information on the prevalence of overweight and obesity among adults in Southwestern China and important reference significant for preventing, screening, and controlling overweight and obesity in this vast population.

## Data Availability

The raw data supporting the conclusions of this article will be made available by the authors, without undue reservation.
